# Pharmacokinetics and toxicity of carboplatin used for hyperthermic intraperitoneal chemotherapy (HIPEC) in treatment of epithelial ovarian cancer

**DOI:** 10.1515/pp-2020-0137

**Published:** 2020-09-07

**Authors:** Mette Schou Mikkelsen, Jan Blaakaer, Lone Kjeld Petersen, Luise Gram Schleiss, Lene Hjerrild Iversen

**Affiliations:** Department of Gynecology and Obstetrics, Aarhus University Hospital, Aarhus, Denmark; Department of Clinical Research, University of Southern Denmark, Odense, Denmark; Open Patient Explorative Data Network, Department of Clinical Research, University of Southern Denmark, Odense, Denmark; Discovery and Development PKPD, Novo Nordisk A/S, Maaloev, Denmark; Department of Surgery, Aarhus University Hospital, Aarhus, Denmark; Department of Gynecology and Obstetrics, Odense University Hospital, Odense, Denmark

**Keywords:** carboplatin, hyperthermic intraperitoneal chemotherapy, ovarian epithelial cancer, pharmacokinetics

## Abstract

**Objectives:**

Carboplatin is frequently used in various doses for hyperthermic intraperitoneal chemotherapy (HIPEC) in the treatment of epithelial ovarian cancer (EOC) although its pharmacokinetics, including focus on the perfusion time, has not been evaluated when used in modern era cytoreductive surgery (CRS). The aim was to evaluate the pharmacokinetics and hematological toxicity of carboplatin used for HIPEC with a perfusion time of 90 min.

**Methods:**

Fifteen patients with stage III–IV primary EOC received CRS and 90 min of HIPEC with carboplatin at dose 800 mg/m^2^. For the pharmacokinetic analysis, perfusate and blood samples were obtained during HIPEC and up to 48 h after HIPEC (blood only). Hematological toxicity within 30 days was graded according to Common Terminology Criteria for Adverse Events. Severe toxicity (grades 3–5) is reported.

**Results:**

Mean maximum concentration of carboplatin was 12 times higher in perfusate than plasma (mean CmaxPF=348 µg/mL (range: 279–595 µg/mL) versus mean CmaxPL=29 µg/mL (range: 21–39 µg/mL)). Mean terminal half-life of carboplatin in perfusate was 104 min (range: 63–190 min) and mean intraperitoneal-to-plasma area under the concentration-time curve (AUC) ratio was 12.3 (range: 7.4–17.2). Two patients (13%) had grade 3 neutropenia within 30 days. No grade 4–5 hematological toxicities were identified.

**Conclusions:**

Carboplatin has a favorable pharmacokinetic profile for 90 min HIPEC administration, and the hematological toxicity was acceptable at dose 800 mg/m^2^. Large interindividual differences were found in the pharmacokinetic parameters, making risk of systemic exposure difficult to predict.

## Introduction

Ovarian cancer is categorized as a peritoneal surface malignancy due to its most common clinical presentation in advanced stages, which is characterized by diffuse peritoneal metastatic spreading. Advanced stage ovarian cancer corresponds to the International Federation of Gynecology and Obstetrics stage III–IV (FIGO stage III–IV). Globally, 240,000 women are diagnosed with ovarian cancer every year, and epithelial carcinomas account for approximately 90% of all cases of ovarian cancer [1]. In the subsequent text, the term “epithelial ovarian cancer (EOC)” will refer to all epithelial cancers that arise in the ovary or fallopian tube, as well as the histologically similar primary peritoneal cancers, as these are commonly considered identical diseases.

Standard treatment of primary advanced stage EOC consists of “upfront”’ cytoreductive surgery (CRS) combined with systemic platinum-based and taxane chemotherapy [2]. Neoadjuvant chemotherapy followed by “interval” CRS and postoperative chemotherapy is used for patients unsuitable for upfront CRS due to excessive tumor burden or poor general condition. Unfortunately, even if primary treatment with CRS and systemic chemotherapy leads to disease control, a major challenge in ovarian cancer treatment is disease relapse, which most often occurs within the first 2 years. In an attempt to prevent or postpone disease relapse, hyperthermic intraperitoneal chemotherapy (HIPEC) has been implemented in some surgical centers for treatment of EOC, often in the form of a clinical trial [3]. Whether HIPEC has a role in the treatment of EOC is still heavily debated [4], [5], [6], [7].

HIPEC consists of intraoperative perfusion of the peritoneal cavity with a heated solution containing cytotoxic agents, performed immediately after the surgical procedure. The rationale for local application is to expose any cancer cells directly to the cytotoxic drug independently of the blood perfusion of the area.

Currently, carboplatin is the standard platinum-based drug of choice used for first-line systemic treatment of EOC. It has a high molecular weight, is highly stable, water-soluble, and is rapidly cleared from the systemic circulation [8], which in theory also makes it a suitable drug for HIPEC. Carboplatin has been described used in HIPEC at a dose range of 400–1200 mg/m^2^ [9], [10], [11], [12]. Whereas several studies have evaluated the pharmacokinetics of other platinum-based drugs used in HIPEC such as cisplatin and oxaliplatin [13], [14], [15], [16], [17], only one small phase I study with six patients published in 1999 by Steller et al. [9] evaluated the pharmacokinetics of carboplatin. Additionally, EOC surgery has become more and more extensive during the last two decades in an attempt to achieve complete cytoreduction. Whether this more aggressive surgical approach affects the absorption of carboplatin from the perfusate into the systemic circulation has not yet been evaluated. Furthermore, most centers perform carboplatin HIPEC with a perfusion time of 90 min, but the rationale for using this perfusion time is often not described [9], [10], [11], [18]. It has not yet been sufficiently evaluated whether 90 min is the appropriate perfusion time. If more than half of the dose is absorbed after e.g., 60 min, one can question whether further 30 min of perfusion is recommendable.

Our primary study aim was to evaluate the local and systemic pharmacokinetics of carboplatin 800 mg/m^2^ used in HIPEC with a perfusion time of 90 min as part of upfront or interval CRS in the treatment of primary EOC and evaluate any relation between the extent of peritonectomy and systemic exposure to carboplatin. The secondary aim was to evaluate hematological toxicity within 30 days after surgery.

## Materials and methods

### Study design and setting

A prospective observational cohort study was conducted at Department of Gynecology and Obstetrics, Aarhus University Hospital, Denmark, from January 2017 to January 2019. CRS with HIPEC was introduced in our department in January 2016 as a feasibility study in 25 patients [19]. The last 15 patients in the feasibility study were additionally enrolled in this pharmacokinetic study in order to document the pharmacokinetic profile for the dose and the perfusion time used, see below.

### Patients

Eligible study participants were women with FIGO stage III–IV primary EOC allocated for either upfront or interval CRS. Obligate inclusion criteria were (a) age 18–75 years; (b) American Society of Anesthesiologists scores I–II (ASA scores I–II); (c) normal biochemical tests of bone marrow, kidney, and liver function; (d) completeness of cytoreduction score 0 (CC–0) [20], defined as no macroscopic tumor nodules remained after surgery; and (e) no psychiatric illness or social conditions making patients unable to follow study requirements and/or give informed consent. Patients with stage IV were only included if they had resectable metastases within the abdominal cavity or abdominal wall, or if there was complete remission of extra-abdominal metastatic disease after three series of neoadjuvant chemotherapy.

All patients had a midline incisional laparotomy from the symphysis to the xiphoid process. At the onset of surgery, the extent of disease was evaluated using the peritoneal cancer index (PCI) developed by Jaquet and Sugarbaker [21]. Three gynecologic oncologists specialized in CRS were included in the surgical team. They were assisted by a colorectal surgeon and/or a surgeon experienced in upper abdominal surgery whenever resection of bowel and/or upper gastrointestinal organ resection was performed. Patients who received both upper and lower abdominal peritonectomy procedures were categorized as having a major peritonectomy procedure. If only a lower abdominal peritonectomy procedure was performed, patients were categorized as having a minor peritonectomy procedure.

### HIPEC procedure

**Figure 1:**
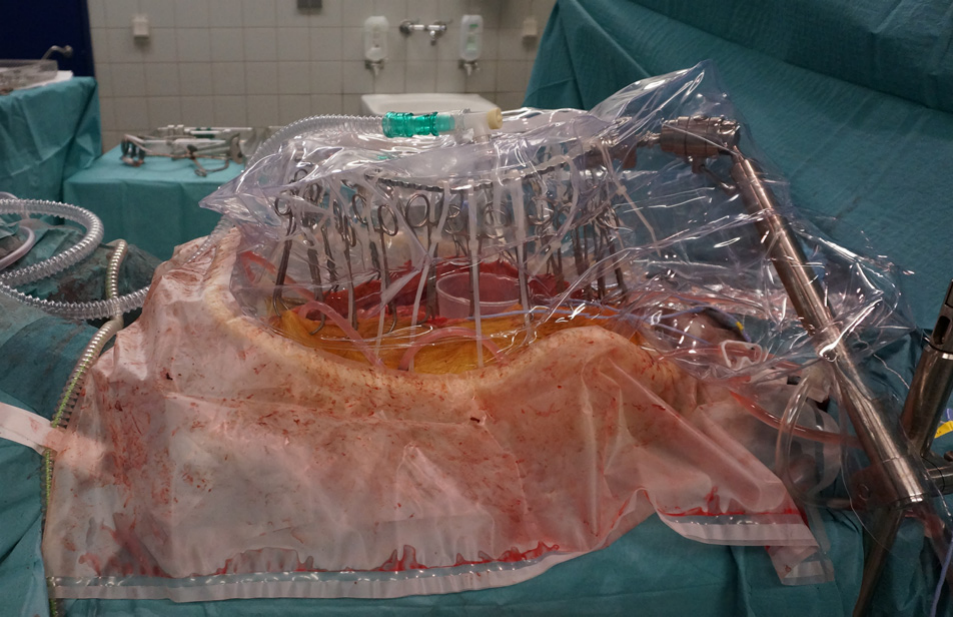
Hyperthermic intraperitoneal chemotherapy (HIPEC) performed with the open abdominal technique.

Following the cytoreductive procedure, HIPEC was performed with an open abdominal technique using the Performer HT® system from RAND (Medolla, Italy). A plastic cover with a slit was used to diminish heat loss from the perfusate and allowed the surgeon’s hand to get access to the abdomen. The setup for the open abdominal technique is shown in Figure 1. Two inflow and three outflow catheters were connected to the perfusion system, and 5000 mL of 0.9% isotonic sodium chloride was heated and used as carrier solution. When perfusate temperature was stable at 41–42 °C*,* carboplatin 800 mg/m^2^ was added to the perfusate as a single dose via the patient inflow catheters. Two dose escalating phase I studies have already estimated the maximum tolerated dose of carboplatin to be 800 mg/m^2^ and 1000 mg/m^2^, respectively [9], [10], and as HIPEC was a new procedure in our department we chose the lowest of these for safety reasons. Furthermore, a maximum dose of 1600 mg carboplatin was used if the body surface area was more than 2.0 m^2^. Perfusion time was 90 min, as we wanted to evaluate pharmacokinetics at the perfusion time, which was most often used for carboplatin HIPEC. After completion of HIPEC, perfusate was immediately drained from the abdominal cavity, and no further salvage was performed before abdominal wound closing. An abdominal drainage tube was left in Douglas’s pouch for 2 days postsurgery.

### Sample collection, storage, and analysis

Perfusate samples were taken from the joined outflow catheters at 0, 5, 7½, 10, 20, 30, 40, 50, 60, 70, 80, and 90 min of HIPEC. Blood samples were obtained in heparinized tubes at 0, 15, 30, 45, 60, 75, and 90 min of HIPEC and 8, 16, 24, and 48 h after HIPEC. Blood samples were centrifuged at 2000 g for 10 min at 4 °C to separate plasma from the blood cells, and 2 mL plasma samples were obtained for storage.

Perfusate and plasma samples were stored at −80 °C until analysis. Total carboplatin concentrations were determined using liquid chromatography–mass spectrometry, where the lower limit of quantification (LLQ) was 0.01 µg/mL and the coefficient of variation was below 10%. Samples with higher concentrations than the calibration range (0.01–5.00 µg/mL) were diluted and reassayed.

### Pharmacokinetics

Carboplatin pharmacokinetics was assessed by noncompartmental analysis using Phoenix® WinNonlin® version 8.0. The pharmacokinetic parameters calculated were the maximum concentration in perfusate and plasma, CmaxPF and CmaxPL; terminal half-life in perfusate and plasma, T½PF and T½PL; and area under the concentration-time curves (AUCs), using the trapezoidal method: AUC for perfusate in the perfusion period, AUC90PF; AUC for plasma in the perfusion period, AUC90PL; and AUC for plasma in the total sampling period, AUCtotalPL. Individual values for AUC90PF, AUC90PL, and AUCtotalPL were interpreted as a measure of carboplatin exposure. Finally, the intraperitoneal-to-plasma AUC ratio for the 90 min perfusion time was calculated for each patient (AUC90PF/AUC90PL).

Samples with concentration values below LLQ were excluded from the pharmacokinetic analysis.

### Hematological toxicity

Hematological toxicity defined as episodes of leucopenia, neutropenia, thrombocytopenia, and/or anemia within 30 days was evaluated and recorded according to the Common Terminology Criteria for Adverse Events, v4.0 [22].

Blood samples evaluating hemoglobin and white blood cell count with differential and platelets were taken the day before surgery, daily on days 1–7, and on days 14, 21, and 30 after surgery. Anemia and thrombocytopenia registered during surgery and the first postoperative day were not interpreted as hematological toxicity but as a result of the CRS procedure. A further decrease in these parameters within day 1–30 postoperatively was assessed as HIPEC-related hematological toxicity. Severe and life-threatening toxicity (grades 3–5) is reported here.

### Statistical analyses

Demographic and clinical data were prospectively collected and analyzed with descriptive statistics using STATA/IC, version 14. The pharmacokinetic parameters were considered normally distributed when transformed to the logarithmic scale. In the statistical analyses, mean estimates of all pharmacokinetic parameters are presented as geometric means with 95% confidence intervals (95% CIs).

Difference in mean AUC ratio between minor and major peritonectomy groups was explored with Student’s *t*-test. A p-value <0.5 was considered statistically significant.

## Results

### Patients, surgery, and HIPEC

Fifteen patients with EOC were enrolled in the study from January 2017 to January 2019. Six patients were in stage III, and nine patients were in stage IV at the time of diagnosis. Patient characteristics are outlined in Table 1. Four patients received HIPEC with carboplatin as part of upfront CRS and 11 patients as part of interval CRS. Median PCI was 8 (range: 3–22), and the median operative time including the HIPEC procedure was 321 min (range: 260–450 min). Details regarding CRS and the HIPEC procedure are given in Table 2.

**Table 1:** Patient characteristics, n=15.

Characteristic	Median	(Range)
Age, years	56	(43–72)
BMI, kg/m^2^	24	(20–38)

	N	(%)
*ASA*		
ASA I	2	(13.3)
ASA II	13	(86.7)
*Primary tumor site*		
Fallopian tube	5	(33.3)
Ovary	7	(46.7)
Primary peritoneal	3	(20.0)
*Histology*		
High grade serous	12	(80.0)
Clear cell	3	(20.0)
*FIGO stage*		
FIGO III	6	(40.0)
FIGO IV	9	(60.0)

N, number of patients; BMI, body mass index; ASA, American Society of Anesthesiologists score; FIGO, International Federation of Gynecology and Obstetrics.

**Table 2:** CRS and HIPEC details, n=15.

Patient ID	Time of CRS	PCI	Extent of peritonectomy	Duration of surgery including HIPEC (min)	Intraoperative bleeding (mL)	Perfusate volume in abdomen (L)	Body surface area (m^2^)	Carboplatin dose (mg)
1	Interval	8	Minor	317	1700	2.0	2.02	1600^a^
2	Upfront	12	Major	300	750	3.8	1.92	1536
3	Interval	5	Minor	360	1700	1.8	1.42	1136
4	Interval	8	Minor	397	500	3.9	2.16	1600^a^
5	Interval	22	Major	416	1300	2.1	1.76	1408
6	Interval	8	Minor	315	500	2.5	1.93	1544
7	Upfront	17	Major	427	1000	2.4	1.60	1280
8	Upfront	15	Minor	301	770	3.3	1.65	1320
9	Interval	8	Minor	450	500	3.0	1.78	1424
10	Interval	12	Major	321	400	3.4	1.82	1456
11	Upfront	16	Major	346	1000	3.0	1.95	1560
12	Interval	18	Major	390	1500	2.1	2.05	1600^a^
13	Interval	3	Minor	312	500	2.0	2.10	1600^a^
14	Interval	6	Minor	311	650	3.4	1.63	1296
15	Interval	8	Minor	260	250	3.1	1.64	1312
Median		8		321	750	3.0	1.82	1456
Range		3–22		260–450	250–1700	1.8–3.9	1.42–2.16	1136–1600
IQR		8–16		311–397	500–1300	2.1–3.4	1.64–2.02	1312–1600

CRS, cytoreductive surgery; HIPEC, hyperthermic intraperitoneal chemotherapy; PCI, peritoneal cancer index; IQR, interquartile range.

Major peritonectomy: upper and lower abdominal peritonectomies were performed.

Minor peritonectomy: only lower abdominal peritonectomy was performed.

^a^Maximum dose defined in study protocol was 1600 mg equivalent to a body surface area of 2.00 m^2^.

### Pharmacokinetics

In total, 180 perfusate samples and 165 plasma samples were obtained from the 15 patients for the pharmacokinetic analysis. Concentrations in all plasma samples taken 48 h after the start of HIPEC were below LLQ and were excluded from the analysis.

The individual and mean pharmacokinetic parameters of carboplatin are reported in Table 3. Mean maximum concentration of carboplatin was 12 times higher in perfusate than plasma (mean CmaxPF=348 µg/mL (range: 279–595 µg/mL) versus mean CmaxPL=29 µg/mL (range: 21–39 µg/mL)). The mean terminal half-life of carboplatin in perfusate was 104 min (range: 63–190) and 160 (range: 131–193) minutes in plasma. Only in two patients (13.3%), the terminal half-life of carboplatin in perfusate was shorter than the 90 min perfusion time. The mean intraperitoneal-to-plasma AUC ratio was 12.3 (range: 7.4–17.2), and there was no statistically significant difference between the two peritonectomy groups, as the ratio between the mean intraperitoneal-to-plasma AUC ratio in minor peritonectomy group (n=9) and major peritonectomy group (n=6) was 1.00 (p=0.99, 95% CI: 0.75–1.3).

**Table 3:** Carboplatin pharmacokinetics, n=15.

Patient ID	CmaxPF (µg/mL)	CmaxPL (µg/mL)	T½PF (min)	T½PL (min)	AUC90PF (min × µg/mL)	AUC90PL (min × µg/mL)	AUCtotalPL (min × µg/mL)	AUC ratio^a^
1	365	23	123	131	25,865	1,572	5,361	16.5
2	349	27	92	153	22,920	1,956	6,427	11.7
3	279	21	190	170	21,643	1,431	5,120	15.1
4	595	39	63	162	34,122	2,695	10,028	12.7
5	360	32	102	170	24,953	2,032	8,805	12.3
6	407	27	132	173	28,909	1,682	6,451	17.2
7	290	32	106	140	19,956	2,162	6,449	9.2
8	293	29	115	181	20,967	1,955	8,014	10.7
9	297	33	108	147	20,667	2,134	7,330	9.7
10	322	29	96	164	21,813	1,928	6,936	11.3
11	393	29	109	154	26,916	1,928	6,328	14.0
12	400	28	112	182	28,384	1,746	7,310	16.3
13	354	28	98	193	24,304	1,794	6,865	13.5
14	297	32	76	157	17,671	2,384	7,662	7.4
15	318	27	90	134	20,225	1,828	5,850	11.1
Mean^b^	348	29	104	160	23,612	1,926	6,892	12.3
CI_95%_	312–387	27–31	91–120	150–170	21,446–25,997	1,764–2,102	6,247–7,604	10.8–14.0
Range	279–595	21–39	63–190	131–193	17,671–34,122	1,431–2,695	5,120–10,028	7.4–17.2
IQR	297–393	27–32	92–115	147–173	20,667–26,916	1,746–2,134	6,328–7,662	10.7–15.1

Cmax, maximum concentration; PF, perfusate; PL, plasma; T½, terminal half-life; AUC90, area under concentration-time curve during perfusion time; AUCtotal, area under concentration-time curve for total sampling period; CI_95%_, 95% confidence interval; IQR, interquartile range.

^a^AUC90PF/AUC90PL.

^b^Geometric mean.

**Figure 2:**
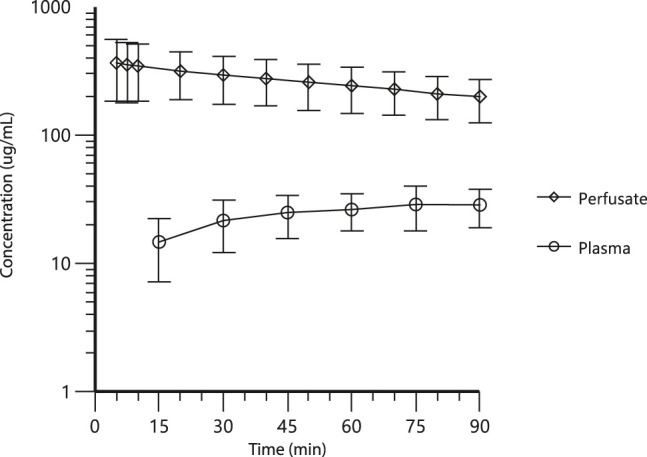
Mean carboplatin concentration µg/mL with standard errors measured in perfusate and plasma during hyperthermic intraperitoneal chemotherapy (HIPEC) perfusion, n=15 patients.

**Figure 3:**
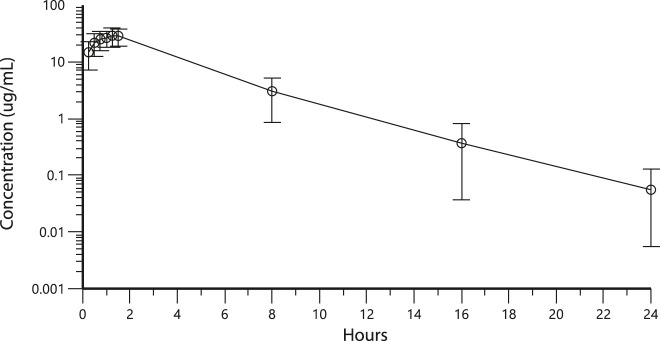
Mean carboplatin concentration µg/mL with standard errors measured in perfusate and plasma during hyperthermic intraperitoneal chemotherapy (HIPEC) perfusion and in plasma at 8,16 and 24 h after the start of HIPEC, n=15 patients.

The mean carboplatin concentrations in perfusate and plasma during HIPEC perfusion and in plasma 8, 16, and 24 h after the start of HIPEC are displayed in Figures 2 and 3, respectively. Figure 1 illustrates that despite high carboplatin concentrations being measured in the perfusate during the total perfusion period, plasma carboplatin concentrations increased only slightly and were around eight times lower even after 90 min of hyperthermic perfusion.

### Hematological toxicity

No deaths or grade 4 (life-threatening) hematological toxicities were identified. Two patients (13%), both of whom had undergone interval CRS, had transient episodes of grade 3 neutropenia (0.5 × 10^9^/L<neutrophil count<1.0 × 10^9^/L) around 9–14 days after surgery. No episodes of grade 3 thrombocytopenia (25.0 × 10^9^/L<platelet count<50.0 × 10^9^/L) or grade 3 anemia (hemoglobin<4.9 mmol/L) were observed within 30 days.

## Discussion

This study evaluated the pharmacokinetics of carboplatin at dose 800 mg/m^2^ used for 90 min HIPEC in 15 patients with EOC undergoing modern era CRS. It was found that carboplatin has a favorable pharmacokinetic profile for HIPEC administration, as high intra-abdominal concentrations of carboplatin were obtained during the entire perfusion with a single-dose administration, and the systemic uptake was limited. Also, no statistical significant relation between the extent of peritonectomy and the systemic absorption of carboplatin was found.

The magnitude of the intraperitoneal-to-plasma AUC ratio reflects the rate of carboplatin clearance from the peritoneal cavity relative to the clearance of carboplatin from the systemic circulation. The mean intraperitoneal-to-plasma AUC ratio in our study corresponds well to the intraperitoneal-to-plasma AUC ratios reported from phase I studies with intraperitoneal administration of carboplatin, which is described to be in the range of 10–18 [8], [23], [24], [25]. Several individual physiological parameters can affect clearance from both compartments such as the size of the diffusion area, blood drainage to the peritoneal surfaces, and kidney function, which the broad range of the individual intraperitoneal-to-plasma AUC ratios in our study (7.4–17.2) clearly illustrates. Variable drug absorption and clearance make the prediction of systemic exposure highly uncertain. Under ideal conditions, an estimation of the expected drug absorption and drug clearance should be made preoperatively for each patient to individualize drug dosage.

The peritoneal-plasma barrier prevents direct diffusion of the cytotoxic drug between blood and the peritoneal cavity. This is a complex physiologic barrier formed by the tissue surrounding the peritoneal space. The peritoneum consists of a single layer of mesothelial cells overlying several layers of connective tissue. The peritoneum plays a major role in secretion of lubricants and generation of local immunological responses, but it does not provide a significant barrier to drug diffusion. The major resistance to drug diffusion from the peritoneal cavity to the systemic circulation consists of the capillary endothelium and the cell-matrix system surrounding the vessel in the subperitoneal tissue [26]. This is probably the main reason why stripping of large surfaces of the peritoneum, which is common in CRS, does not seem to affect transport of the intraperitoneal delivered drug into the systemic circulation. Data from other studies and our data support this notion [27], [28].

Several parameters can affect the pharmacokinetics of the drug used for HIPEC, such as the properties of the carrier solution, volume of the perfusate, temperature level, duration of HIPEC, and the technique used (open or closed) [29]. A long perfusate terminal half-life of carboplatin (104 min) was found in this study compared to cisplatin, which has a range of 43–70 min [14], [15]. This can partly be explained by the physical properties of the carrier solution used in this study. For instance, isotonic sodium chloride has a low molecular weight and is absorbed faster from the peritoneal cavity than carboplatin, which may lead to a higher concentration of carboplatin in the carrier solution, and thereby affect the perfusate terminal half-life. The long terminal half-life also reflects that carboplatin stability is high even under hyperthermic conditions (temperature: 41–42 °C). Based on this result showing a long perfusate terminal half-life of carboplatin, we recommend a single-dose administration of carboplatin when used for HIPEC, as high concentrations of carboplatin were found in the perfusate during the entire 90 min of perfusion in all patients. Also, the perfusion time of 90 min seems to be appropriate, as the absorption rate from the perfusate was low in the majority of our patients.

Minimal hematological toxicity after HIPEC with carboplatin at dose 800 mg/m^2^ was observed in this study and was within the range described by other authors [9], [10]. Steller et al. [9]found that dose-limiting hematological toxicity was observed at carboplatin dose 1200 mg/m^2^ and was associated with a carboplatin plasma AUC of 11 min × mg/mL. This corresponds well with our findings, as none of our patients had carboplatin plasma AUC values above this threshold value. Both patients with isolated transient grade 3 neutropenia had interval CRS with a minor peritonectomy procedure (Patient IDs 9 and 14 in Tables 2–3). They had low intraperitoneal-to-plasma AUC ratios (7.4 and 9.7), and AUC90PL and AUCtotalPL were above the mean value for both patients. The combination of these pharmacokinetic parameters and increased vulnerability to bone marrow suppression because the patients had already received neoadjuvant chemotherapy could be possible explanations. It is, however, important to emphasize that neither of the two patients had episodes of febrile neutropenia, changes in other hematological parameters, or other complications. Hypothetically, it seems natural to lower the dosage of the chemotherapy used in HIPEC as part of interval CRS, if the patient during neoadjuvant chemotherapy treatments had experienced severe bone marrow suppression, but it is beyond the scope of the present study.

One strength of the present study is that the pharmacokinetics of carboplatin was evaluated in 15 patients, given the same body surface dosage of carboplatin (800 mg/m^2^) and a fixed volume of the carrier solution, which clearly visualizes the great interindividual variation of the pharmacokinetic parameters. The prospective study design including the systematic biochemical evaluation of the patients is another strength. The total concentration of carboplatin was measured in the plasma samples (protein-bound + free carboplatin) but the concentration of ultrafilterable carboplatin (free carboplatin) was not measured, which is a potential limitation of this study seen in a clinically perspective because only free platinum species are considered cytotoxic [30]. Another limitation is that HIPEC-induced severe anemia might have been masked because administration of red blood cell transfusion in the perioperative and postoperative period was allowed if the patient had symptoms of anemia that restricted postoperative mobilization of the patient.

## Conclusions

In conclusion, our study demonstrates that carboplatin has a favorable pharmacokinetic profile for HIPEC administration after an extensive CRS procedure, but large interindividual differences were found in the pharmacokinetic parameters. Based on our pharmacokinetic analysis, we recommend a single-dose administration of carboplatin and a perfusion time of 90 min. No relation between the extent of peritonectomy and systemic exposure of carboplatin was found, and the hematological toxicity was acceptable at a carboplatin dose of 800 mg/m^2^. We recommend that future pharmacokinetic studies focus on the interindividual differences in drug absorption during HIPEC, so a clinical tool that would make possible calculation of an individualized dose based on the presumed uptake of the drug by the individual patient can be developed.

## References

[1] Webb PM , Jordan SJ . Epidemiology of epithelial ovarian cancer. Best Pract Res Clin Obstet Gynaecol 2017;41:3–14. 10.1016/j.bpobgyn.2016.08.006. 27743768

[2] Orr B , Edwards RP . Diagnosis and treatment of ovarian cancer. Hematol Oncol Clin N Am 2018;32:943–64. 10.1016/j.hoc.2018.07.010. 30390767

[3] Morano WF , Khalili M , Chi DS , Bowne WB , Esquivel J . Clinical studies in CRS and HIPEC: trials, tribulations, and future directions-a systematic review. J Surg Oncol 2018;117:245–59. 10.1002/jso.24813. 29120491 PMC6692902

[4] Vergote I , Harter P , Chiva L . Is there a role for intraperitoneal chemotherapy, including HIPEC, in the management of ovarian cancer?. J Clin Oncol 2019;37:2420–3. 10.1200/jco.19.00091. 31403870

[5] Zivanovic O , Chi DS , Filippova O , Randall LM , Bristow RE , O’Cearbhaill RE . It’s time to warm up to hyperthermic intraperitoneal chemotherapy for patients with ovarian cancer. Gynecol Oncol 2018;151:555–61. 10.1016/j.ygyno.2018.09.007. 30249527 PMC6684262

[6] Lim MC , Chang S-J , Yoo HJ , Nam B-H , Bristow R , Park S-Y . Randomized trial of hyperthermic intraperitoneal chemotherapy (HIPEC) in women with primary advanced peritoneal, ovarian, and tubal cancer. J Clin Oncol 2017;35:5520. 10.1200/jco.2017.35.15_suppl.5520.

[7] van Driel WJ , Koole SN , Sikorska K , van Leeuwen JHS , Schreuder HWR , Hermans RHM , . Hyperthermic intraperitoneal chemotherapy in ovarian cancer. N Engl J Med 2018;378:230–40. 10.1056/nejmoa1708618. 29342393

[8] Elferink F , van der Vijgh WJ , Klein I , ten Bokkel Huinink WW , Dubbelman R , McVie JG . Pharmacokinetics of carboplatin after intraperitoneal administration. Canc Chemother Pharmacol 1988;21:57–60. 10.1007/bf00262740. 3277734

[9] Steller MA , Egorin MJ , Trimble EL , Bartlett DL , Zuhowski EG , Alexander HR , . A pilot phase I trial of continuous hyperthermic peritoneal perfusion with high-dose carboplatin as primary treatment of patients with small-volume residual ovarian cancer. Canc Chemother Pharmacol 1999;43:106–14. 10.1007/s002800050870. 9923815

[10] Lentz SS , Miller BE , Kucera GL , Levine EA . Intraperitoneal hyperthermic chemotherapy using carboplatin: a phase I analysis in ovarian carcinoma. Gynecol Oncol 2007;106:207–10. 10.1016/j.ygyno.2007.03.022. 17498782

[11] Cripe J , Tseng J , Eskander R , Fader AN , Tanner E , Bristow R . Cytoreductive surgery and hyperthermic intraperitoneal chemotherapy for recurrent ovarian carcinoma: analysis of 30-day morbidity and mortality. Ann Surg Oncol 2015;22:655–61. 10.1245/s10434-014-4026-6. 25155402

[12] Argenta PA , Sueblinvong T , Geller MA , Jonson AL , Downs LS , Carson LF , . Hyperthermic intraperitoneal chemotherapy with carboplatin for optimally-cytoreduced, recurrent, platinum-sensitive ovarian carcinoma: a pilot study. Gynecol Oncol 2013;129:81–5. 10.1016/j.ygyno.2013.01.010. 23352917

[13] Cho HK , Lush RM , Bartlett DL , Alexander HR , Wu PC , Libutti SK , . Pharmacokinetics of cisplatin administered by continuous hyperthermic peritoneal perfusion (CHPP) to patients with peritoneal carcinomatosis. J Clin Pharmacol 1999;39:394–401. 10.1177/00912709922007967. 10197298

[14] Cattel L , De Simone M , Passera R , Verlengo MC , Delprino L . Pharmacokinetics of cisplatin in semi-closed hyperthermic peritoneal perfusion (HPP) for treatment of peritoneal carcinomatosis. Anticancer Res 2004;24:2041–5. 15274398

[15] Zivanovic O , Abramian A , Kullmann M , Fuhrmann C , Coch C , Hoeller T , . HIPEC ROC I: a phase I study of cisplatin administered as hyperthermic intraoperative intraperitoneal chemoperfusion followed by postoperative intravenous platinum-based chemotherapy in patients with platinum-sensitive recurrent epithelial ovarian cancer. Int J Cancer 2015;136:699–708. 10.1002/ijc.29011. 24895230

[16] Chalret du Rieu Q , White-Koning M , Picaud L , Lochon I , Marsili S , Gladieff L , . Population pharmacokinetics of peritoneal, plasma ultrafiltrated and protein-bound oxaliplatin concentrations in patients with disseminated peritoneal cancer after intraperitoneal hyperthermic chemoperfusion of oxaliplatin following cytoreductive surgery: correlation between oxaliplatin exposure and thrombocytopenia. Canc Chemother Pharmacol 2014;74:571–82. 10.1007/s00280-014-2525-6. 25053386

[17] Petrillo M , Zucchetti M , Cianci S , Morosi L , Ronsini C , Colombo A , . Pharmacokinetics of cisplatin during open and minimally-invasive secondary cytoreductive surgery plus HIPEC in women with platinum-sensitive recurrent ovarian cancer: a prospective study. J Gynecol Oncol 2019;30:e59. 10.3802/jgo.2019.30.e59. 31074245 PMC6543101

[18] Rettenmaier MA , Mendivil AA , Abaid LN , Brown JV , Micha JP , Wilcox AM , . The feasibility of administering varying high-dose consolidation hyperthermic intraperitoneal chemotherapy with carboplatin in the treatment of ovarian carcinoma. Arch Gynecol Obstet 2015;291:1381–6. 10.1007/s00404-014-3590-0. 25516177

[19] Mikkelsen MS , Christiansen T , Petersen LK , Blaakaer J , Iversen LH . Morbidity after cytoreductive surgery and hyperthermic intraperitoneal chemotherapy with carboplatin used for ovarian, tubal, and primary peritoneal cancer. J Surg Oncol 2019;120:550–7. 10.1002/jso.25603. 31267569

[20] Sugarbaker PH . Management of peritoneal-surface malignancy: the surgeon’s role. Langenbeck’s Arch Surg 1999;384:576–87. 10.1007/s004230050246. 10654274

[21] Jacquet P , Sugarbaker PH . Clinical research methodologies in diagnosis and staging of patients with peritoneal carcinomatosis. Cancer Treat Res 1996;82:359–74. 10.1007/978-1-4613-1247-5_23. 8849962

[22] National Cancer Institute. Common Terminology criteria for Adverse Events (CTCA) version 4.0 2009. Available from https://evs.nci.nih.gov/ftp1/CTCAE/CTCAE_4.03/CTCAE_4.03_2010-06-14_QuickReference_5x7.pdf Accessed 31 July 2020.

[23] DeGregorio MW , Lum BL , Holleran WM , Wilbur BJ , Sikic BI . Preliminary observations of intraperitoneal carboplatin pharmacokinetics during a phase I study of the Northern California Oncology Group. Canc Chemother Pharmacol 1986;18:235–8. 10.1007/bf00273393. 3542268

[24] McClay EF , Goel R , Andrews P , Gorelick S , Kirmani S , Kim S , . A phase I and pharmacokinetic study of intraperitoneal carboplatin and etoposide. Br J Cancer 1993;68:783–8. 10.1038/bjc.1993.428. 8398708 PMC1968622

[25] Jandial DA , Brady WE , Howell SB , Lankes HA , Schilder RJ , Beumer JH , . A phase I pharmacokinetic study of intraperitoneal bortezomib and carboplatin in patients with persistent or recurrent ovarian cancer: an NRG Oncology/Gynecologic Oncology Group study. Gynecol Oncol 2017;145:236–42. 10.1016/j.ygyno.2017.03.013. 28341300 PMC5706109

[26] Flessner MF . The transport barrier in intraperitoneal therapy. Am J Physiol Renal Physiol 2005;288:433–42. 10.1152/ajprenal.00313.2004. 15692055

[27] de Lima Vazquez V , Stuart OA , Mohamed F , Sugarbaker PH . Extent of parietal peritonectomy does not change intraperitoneal chemotherapy pharmacokinetics. Canc Chemother Pharmacol 2003;52:108–12. 10.1007/s00280-003-0626-8. 12759776

[28] Rubin J , Jones Q , Planch A , Bower JD . The minimal importance of the hollow viscera to peritoneal transport during peritoneal dialysis in the rat. ASAIO transactions 1988;34:912–5. 10.1097/00002480-198804000-00009. 3064790

[29] Kusamura S , Dominique E , Baratti D , Younan R , Deraco M . Drugs, carrier solutions and temperature in hyperthermic intraperitoneal chemotherapy. J Surg Oncol 2008;98:247–52. 10.1002/jso.21051. 18726886

[30] BC Cancer Agency Cancer Drug Manual Available from http://www.bccancer.bc.ca/drug-database-site/Drug%20Index/Carboplatin_monograph_1Jan2014.pdf Accessed 31 July 2020 ..

